# The complete chloroplast genome sequence of spleen amaranth (*Amaranthus dubius* Mart. ex Thell., Amaranthaceae)

**DOI:** 10.1080/23802359.2021.1992318

**Published:** 2021-10-23

**Authors:** Xin-Yan Xu, Jing Yan, Hui-Ru Li, Yu-Qing Feng, Zhe-Chen Qi, Xiao-Ling Yan

**Affiliations:** a Eastern China Conservation Centre for Wild Endangered Plant Resources, Shanghai Chenshan Botanical Garden, Shanghai, China; b Zhejiang Province Key Laboratory of Plant Secondary Metabolism and Regulation, College of Life Sciences and Medicine, Zhejiang Sci-Tech University, Hangzhou, China; c Shaoxing Academy of Biomedicine of Zhejiang Sci-Tech University, Shaoxing, China

**Keywords:** *Amaranthus dubius*, chloroplast genome, naturalized, phylogenetic

## Abstract

*Amaranthus dubius* is a leafy vegetable widely cultivated in Asia and Africa. The complete chloroplast genome of *Amaranthus dubius* was sequenced and assembled in this study. The complete chloroplast genome is 150,520 bp. A total of 130 genes were identified, including 85 protein-coding genes, eight rRNA genes, and 37 tRNA genes. The overall GC content of this genome was 36.6%. The phylogenetic tree based on 10 chloroplast genomes in Amaranthaceae supports that *A. dubius* is sister to *A. hypochondriacus* and *A. caudatus*.

*Amaranthus dubius* Mart. ex Thell. 1912 (Amaranthaceae), an annual herb native to tropical America, is widely cultivated as a green vegetable and considered a medicinal herb in many Asian and African countries (Achigan-Dako et al. 2014; Alegbejo 2014). Studies have shown that the leaves of *A. dubius* can be used as complement dietary for rice, wheat, and corn proteins (Rodríguez et al. 2011). For the past decades, it has escaped from cultivation and now it is considered naturalized throughout the tropical and subtropical regions of Europe, Asia, and Africa (Wang et al. 2015). Despite being used as an important vegetable, little molecular genetic information of this species has been reported. A recent study showed that there is a disagreement between the concatenated chloroplast genes and nuclear datasets in the placement of the species, which indicates a possible complex evolutionary history of this species (Waselkov et al. 2018). Additionally, *A. dubius* is the only known allotetraploid *Amaranthus* species. To further study its species origin, we assembled and reported the complete chloroplast genome of *A. dubius* here for the first time. We hope it will provide valuable genetic resources for comparative genomic studies in resolving the evolution and underlying genetic questions in *Amaranthus*.

The *A. dubius* individual was collected from Fuzhou, China (GPS: E 116°20′25.08″, N 26°50′37.32″). DNA was extracted from its dried specimen leaves using Plant Genomic DNA Kit (Tiangen, Beijing, Co., Ltd., Beijing, China). The specimen and extracted DNA was deposited at Shanghai Chenshan Herbarium (CSH), Shanghai Chenshan Botanical Garden (http://www.csnbgsh.cn) under the voucher number RQHD03140 (collected by Xiao-Ling Yan: sx_yxl@163.com). The plastome sequences were generated using the Illumina HiSeq 2500 platform (Illumina Inc., San Diego, CA). In total, about 16 million high-quality clean reads (150 bp PE read length) were generated with adaptors trimmed. Aligning, assembly, and annotation were conducted by GetOrganelle (Jin et al, 2020), GeSeq (Tillich et al. 2017), and GENEIOUS v11.1.5 (Biomatters Ltd., Auckland, New Zealand).

The full length of *A. dubius* chloroplast sequence (GenBank accession no. MZ397802) is 150,520 bp. It is comprised by a large single copy region (LSC with 83,869 bp), a small single copy region (SSC with 17,947 bp), and two inverted repeat regions (IR with 24,352 bp). The GC content of *A. dubius* chloroplast genome was 36.6% and the GC contents of the LSC, SSC, and IR regions are 34.5%, 30.3%, and 42.6%. The genome (85 protein-coding genes, eight rRNA genes, and 37 tRNA genes) contained 130 genes. Seventeen genes had two copies, which were comprised of six PCG genes (*ndh*B, *rpl*2, *rpl*23, *rps*12, *rps*7, *ycf*2), seven tRNA genes (*trn*A-UGC, *trn*I-CAU, *trn*I-GAU, *trn*L-CAA, *trn*N-GUU, *trn*R-ACG, *trn*V-GAC), and all four rRNA species (*rrn*16, *rrn*23, *rrn*4.5, *rrn*5). In the genome, seven protein-coding genes (*rps*16, *atp*F, *rpo*C1, *pet*B, *rpl*16, *ndh*B, *ndh*A) had one intron, and three protein-coding genes (*rps*12, *ycf*3, *clp*P) contained two introns.

To analyze the phylogenetic placement of *A. dubius* in *Amaranthus*, chloroplast genomes sequences of additional eight *Amaranthus* species and *Alternanthera philoxeroides* as outgroup were obtained from NCBI. The sequence alignment was conducted by MAFFT v7.450 (Katoh and Standley 2013), of which parameter used the default. Based on TVM + F+G4 model and 5000 bootstrap replicates, the maximum-likelihood (ML) analysis was performed by using IQTREE v2.0.6 (Nguyen et al. 2015). The result indicates that *A. dubius* is sister to a clade formed by *A. hypochondriacus* and *A. caudatus* (Figure 1). We hope the complete chloroplast genome of *A. dubius* will provide necessary genetic resource and background data for further phylogenetic study of the Amaranthaceae.

**Figure 1.**
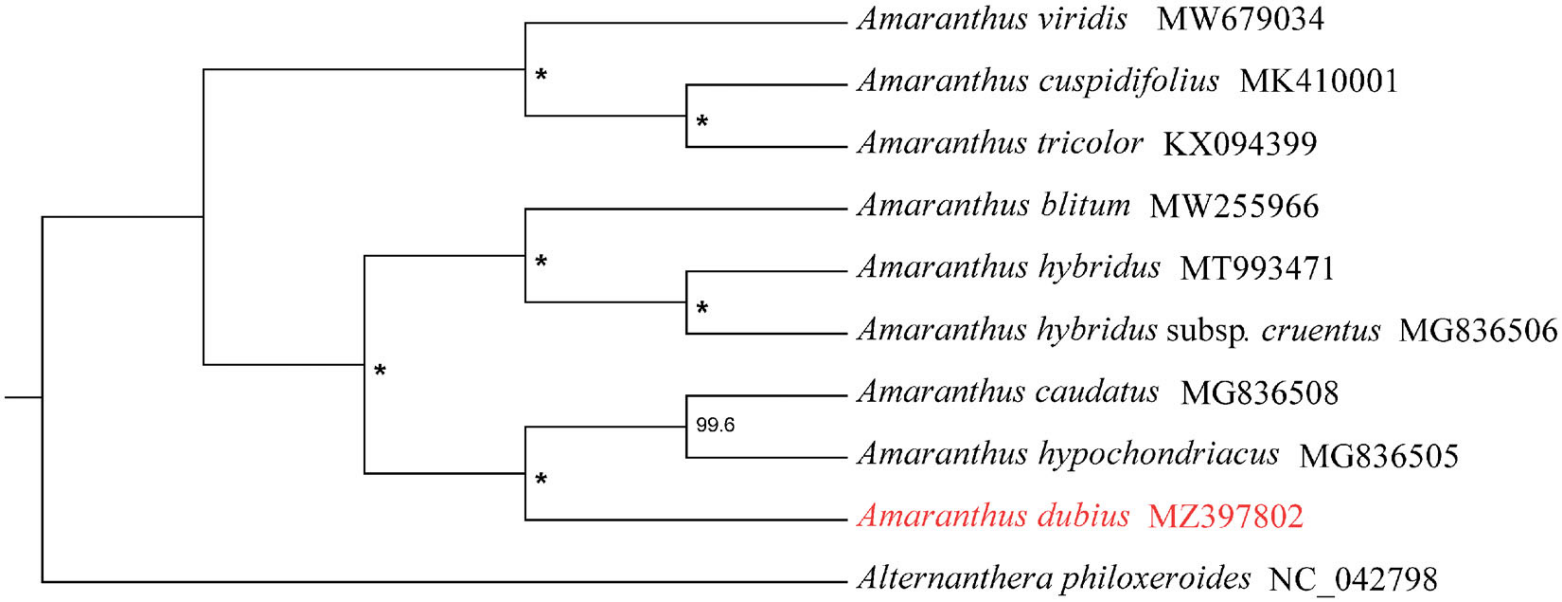
Phylogeny of *Amaranthus* based on complete chloroplast genomes (accession numbers were listed behind each taxon; statistical support values were shown on nodes; *a 100% bootstrap value).

## Data Availability

The genome sequence data that support the findings of this study are openly available in GenBank of NCBI (https://www.ncbi.nlm.nih.gov) under the accession no. MZ397802. The associated BioProject, SRA, and Bio-Sample numbers are PRJNA741594, SRR14920259, and SAMN19882390, respectively. The DNA matrix and phylogenetic tree that support the findings of this study are openly available on figshare: https://doi.org/10.6084/m9.figshare.16530771.
